# Minor differences in haplotype frequency estimates can produce very large differences in heterogeneity test statistics

**DOI:** 10.1186/1471-2156-8-38

**Published:** 2007-06-27

**Authors:** David Curtis, Ke Xu

**Affiliations:** 1 Academic Department of Psychiatry, Queen Mary's School of Medicine and Dentistry, London E1 1BB, UK; 2 Laboratory of Neurogenetics, National Institute on Alcohol Abuse and Alcoholism, 5625 Fishers Lane, Room 3S32, Bethesda, MD 20892-9412, USA

## Abstract

**Background:**

Tests for association between a haplotype and disease are commonly performed using a likelihood ratio test for heterogeneity between case and control haplotype frequencies. Using data from a study of association between heroin dependence and the DRD2 gene, we obtained estimated haplotype frequencies and the associated likelihood ratio statistic using two different computer programs, MLOCUS and GENECOUNTING. We also carried out permutation testing to assess the empirical significance of the results obtained.

**Results:**

Both programs yielded similar, though not identical, estimates for the haplotype frequencies. MLOCUS produced a p value of 1.8*10^-15 ^and GENECOUNTING produced a p value of 5.4*10^-4^. Permutation testing produced a p value 2.8*10^-4^.

**Conclusion:**

The fact that very large differences occur between the likelihood ratio statistics from the two programs may reflect the fact that the haplotype frequencies for the combined group are not constrained to be equal to the weighted averages of the frequencies for the cases and controls, as they would be if they were directly observed rather than being estimated. Minor differences in haplotype frequency estimates can result in very large differences in the likelihood ratio statistic and associated *p *value.

## Background

We wish to point out a serious and previously undescribed problem with using heterogeneity testing to test for differences in haplotype frequencies between cases and controls. As implemented in several applications [1-3] a likelihood ratio statistic (LRS) is calculated as 2(L_CASE_+L_CONTROL_-L_COMBINED_), where the relevant log likelihoods are produced using maximum likelihood estimates of the haplotype frequencies. Although this approach is reasonable in theory, severe problems can arise in practice if the likelihood maximisation process is less than perfect. Although the estimation-maximisation (EM) method usually performs well, it is known that it is not guaranteed always to find a global maximum. In many circumstances small errors in numerical estimates do not have practical signficance. However we have discovered that in the context of heterogeneity testing apparently trivial differences in estimating the haplotype frequency parameters can have very large impacts on the associated likelihoods and LRS values.

## Results

In Table 1 we present the haplotype frequency estimates and associated log likelihoods and LRS values obtained from genotyping DRD2 polymorphisms in 503 subjects with heroin dependence and 336 controls [4] using MLOCUS [2] and GENECOUNTING [3,5]. It can be seen that although there are only slight variations in the frequency estimates there is a very large difference between the LRS values obtained leading to statistical significance levels of either p = 1.8*10^-15 ^or p = 5.4*10^-4^. An empirical test of significance using permutation testing of the GENECOUNTING result produced a value of *p *= 2.8*10^-4^.

**Table 1:** Table showing haplotype frequency estimates and likelihood ratio statistics obtained from MLOCUS and GENECOUNTING for polymorphisms around DRD2

Haplotype	MLOCUS			GENECOUNTING		
	Cases	Controls	Combined	Cases	Controls	Combined

111111	0.005	0.000	0.011	0.021	0.000	0.012
111112	0.006	0.007	0.007	0.008	0.007	0.008
111121	0.499	0.470	0.480	0.482	0.472	0.479
111221	0.029	0.043	0.034	0.027	0.043	0.033
112111	0.001	0.000	0.002	0.004	0.000	0.002
112112	0.392	0.376	0.385	0.384	0.370	0.379
112121	0.021	0.026	0.021	0.021	0.025	0.023
112122	0.001	0.000	0.000	0.000	0.000	0.000
112221	0.001	0.000	0.001	0.001	0.000	0.001
221112	0.043	0.074	0.055	0.050	0.078	0.061
222112	0.001	0.004	0.003	0.000	0.004	0.002
222212	0.001	0.000	0.001	0.001	0.000	0.000
Log likelihood	-820.072	-573.826	-1458.75	-931.20	-601.13	-1548.8
Likelihood ratio statistic			129.7			33.0
Asymptotic P value assuming 11 *df*			1.8*10^-15^			5.4*10^-4^
Empirical P value from permutation test						2.8*10^-4^

## Discussion

We believe that the magnitude of variation of the LRS values obtained will be surprising to many readers. To begin to understand why this might be the case, let us consider the frequencies estimated for the first haplotype by the MLOCUS program. For cases, controls and the combined sample respectively these consist of 0.005, 0.000 and 0.011. We note that the combined sample actually has an estimated frequency which is higher than that for either cases or controls. Obviously this cannot reflect the real situation but we will acknowledge that numerical approximations and/or the fact that the EM algorithm may not necessarily converge to a true maximum can adequately explain this discrepancy. To gain some appreciation of the effect this will have on calculations of the LRS value it is instructive to consider what would happen if these estimates were applied to actually observed haplotype counts rather than being incorporated into a complex likelihood calculation using weighted probabilities over all possible haplotype configurations, as in fact occurs within these programs. Then we would calculate the contribution to the LRS value for haplotype *i *as LRS_i _= 2*(2*Ncase*ln(Pcase_i_) + 2*Ncontrol*ln(Pcontrol_i_) - 2*Ncombined*ln(Pcombined_i_)), where N represents the number of subjects, 2*N the number of haplotypes and P the estimated frequency in the relevant group. When we do this for the first haplotype we obtain LRS_1 _= 2*(- 27.1+0+83.6) = 113.5. As a contribution to a chi-squared statistic this is obviously extremely large and can be expected to be related to an infinitesimal *p *value. Although the likelihood calculations within the programs do not use the estimated frequencies in this way this simple example shows how small variations in frequency estimates could produce massive changes in the LRS.

If the haplotype frequency estimates were based on observed haplotype counts then they would need to conform to the constraint that the frequency in the combined sample would be equal to the weighted average of the case and control frequencies. However, when haplotype frequencies are estimated from phase-unknown genotypes and are estimated independently in the three groups this constraint need not apply and deviations from it can be seen to lead to surprisingly high LRS values and correspondingly small *p *values. In the present example, the fact that the frequency of one haplotype is estimated to be higher in the combined sample compared to both the cases and controls might draw attention to potential problems but it seems reasonable to expect that much more subtle differences in frequency estimates could still have substantial effects on the statistical inferences drawn from heterogeneity testing.

## Conclusion

Apparently minor differences in estimated frequency can have a surprisingly important impact on the LRS obtained and its associated *p *value. The authors of MLOCUS recommended that permutation testing be used to confirm inferences based on the theoretical distribution of LRS values [2] and we concur with this advice.

## Methods

Haplotype frequencies for 6 SNPs genotyped around the DRD2 locus were estimated in polymorphisms in 503 subjects with heroin dependence and 336 controls [4] using the MLOCUS [2] and using GENECOUNTING and its associated support program, RUNGC [3,5]. The associated log likelihoods were also obtained and the LRS providing evidence for heterogeneity of haplotype frequencies between the samples was calculated as LRS = 2(L_CASE_+L_CONTROL_-L_COMBINED_). For each analysis, an asymptotic *p *value was calculated assuming that there were 11 degrees of freedom, this being one less than the number of haplotype frequencies estimated in each group. An empirical *p *value was also obtained using the RUNGC program to implement sequential Monte Carlo testing [6]. A target number of 10 was set for the number of randomly permuted datasets to yield a LRS as high as that observed in the real dataset.

## Abbreviations

EM – estimation-maximisation

LRS – likelihood ratio statistic

## Authors' contributions

KX reported original analyses, provided raw data and agreed text. DC carried out reanalyses and draughted text. Both authors approved the final version.
